# Examining medication adherence and preferences for a lifestyle intervention among Black and Latinx adults with hypertension: a feasibility study

**DOI:** 10.1186/s40814-021-00930-z

**Published:** 2021-11-22

**Authors:** Emily C. Dougherty, Natasha Burse, Michael Butzner, Hongke Wu, Heather L. Stuckey, Jeroan J. Allison, Yendelela L. Cuffee

**Affiliations:** 1 General Academic Pediatrics, University of Pittsburgh, 3414 Fifth Avenue, Pittsburgh, PA 15213 USA; 2 Department of Public Health Sciences, Penn State College of Medicine, 90 Hope Drive, Academic Support Building, Hershey, PA 17033 USA; 3 Department of Population and Quantitative Health Sciences, University of Massachusetts Medical School, 55 N Lake Ave, Worcester, MA 01655 USA; 4 Epidemiology Program, College of Health Sciences, University of Delaware, Newark, DE USA

**Keywords:** Hypertension, Blacks, African Americans, Latinx, Medication adherence, Health literacy, Technology-based interventions, Behavioral change

## Abstract

**Background:**

Approximately 116.4 million adults in the USA have hypertension, and the rates of uncontrolled hypertension remain higher among racial and ethnic minorities. There is a need for effective interventions that promote healthy behaviors and long-term behavioral change in the management of hypertension. The primary objective of this study was to determine the feasibility of developing a lifestyle intervention that would assess hypertension management and the use of technology among Blacks and Latinx with hypertension. The secondary objective is to explore perceptions of community-based resources for hypertension and preferences for a lifestyle intervention for hypertension among Blacks and Latinx with hypertension.

**Methods:**

In this explanatory mixed-methods study, quantitative data were collected using surveys, participants reported their use of technology and adherence to antihypertensive medication. Participants were Black and Latinx adults with hypertension living in Central Pennsylvania, USA. Qualitative data were obtained from semi-structured interviews and focus groups, and participants were asked about managing hypertension, local resources, and preferences for a behavioral intervention. Data were examined using summary statistics for quantitative data and thematic analysis for qualitative data.

**Results:**

Black and Latinx participants (*n*=30) completed surveys for the quantitative study. The majority (75%) of participants self-reported being confident in managing their medication without help and remembering to take their medication as prescribed. Fewer participants (54.2%) reported using technology to help manage medication. There were 12 participants in the qualitative phase of the study. The qualitative findings indicated that participants felt confident in their ability to manage hypertension and were interested in participating in a lifestyle intervention or program based online. Some participants reported a lack of resources in their community, while others highlighted local and national resources that were helpful in managing high blood pressure.

**Conclusion:**

This study provides important insights on barriers and facilitators for managing hypertension, current use of technology and interest in using technology to manage hypertension, and preferences for future lifestyle interventions among racial and ethnic minorities. This study also provides insights to the health needs and resources available in this community and how future behavioral interventions could be tailored to meet the needs of this community. The findings of this study will be used to inform the tailoring of future lifestyle interventions; specifically, we will include text messaging reminders for medication and to disseminate educational materials related to hypertension and provide resources to connect study participants with local and national resources.

## Key messages regarding feasibility

1) What uncertainties existed regarding the feasibility?

The primary objective of this study was to apply a mixed-methods approach to explore the feasibility of developing a lifestyle and or behavioral intervention using a mobile device or a technology-based platform, and the secondary objective was to determine participant preferences for the delivery of an intervention.

2) What are the key feasibility findings?

Our findings indicated that a mobile or technology-based intervention to deliver health information or manage hypertension might be an engaging approach for promoting healthful behaviors particularly in communities carrying the heaviest burden of cardiovascular disease. Participants also shared barriers and facilitators for managing hypertension.

3) What are the implications of the feasibility findings for the design of the main study?

The findings of this study provide insights into the feasibility of developing a technology-based lifestyle and behavioral intervention for individuals living with and managing hypertension. Study participants shared key barriers to managing hypertension, preferences for in-person vs. online interventions, and topics of interest that might be incorporated into future medication adherence intervention.

## Background

Hypertension is a risk factor for cardiovascular disease including stroke, heart disease, peripheral vascular disease, myocardial infarction, and heart failure [1]. Approximately 116 million adults or every one in three adults in the United States (US) have hypertension [2]. In 2016, 23.2% of Latinx and 40.5% of Blacks had hypertension [3]. The burden of hypertension and cardiovascular disease are highest among racial and ethnic minorities, often individuals living in socioeconomically disadvantaged communities bear the heaviest burden of cardiovascular disease [2, 4].

Blacks have the highest prevalence of hypertension in the US and are at greatest risk for developing complications such as stroke, kidney disease, and end-stage organ damage [5–7]. Latinx typically report a better health status and better health outcomes than Blacks, despite being similar in terms of socioeconomic status [8]. On average, Latinx have the lowest rate of blood pressure control in the US [3]. In comparison to other racial and ethnic groups, Latinx report lower rates of awareness, treatment, and control of hypertension [2].

Hypertension control is achieved through diet, physical activity, and adherence to antihypertensive medications. Approximately half of individuals living with hypertension report being nonadherent to antihypertensive medications, which includes not taking antihypertensive medications, missing doses, and taking partial doses of medications [9]. Medication nonadherence is a persistent problem across all racial and ethnic groups and a barrier to achieving hypertension control. There is a growing need for innovative and effective approaches for promoting medication adherence and approaches that encourage long-term behavioral change.

Technology-based interventions have become an increasingly popular approach for preventing and managing chronic health conditions including type 1 and type 2 diabetes, and hypertension [10–12]. Technology-based interventions (i.e., interventions delivered using a computer, smartphone, text, application, website) generate greater empowerment, quality of life, and treatment adherence [13]. Specifically, mobile health (mHealth) and electronic health (eHealth) are progressively being used in public health for communication, monitoring, and education to improve health outcomes, adherence to chronic disease management, access to care, and service delivery [12]. mHealth and eHealth interventions may be an effective approach for engaging individuals living in socioeconomically disadvantaged communities and individuals managing chronic conditions [13]. James et al. (2017) conducted a systematic review to assess the participation of Blacks in e-Health/m-Health interventions between January 2000 and June 2016 [14] Of the 56 studies included in the review, eight interventions were mHealth. The review concluded there was a low representation of Black participants in e-Health/m-Health research. The authors concluded based on smartphone ownership and usage that mHealth interventions may create new opportunities for community-based research and interventions [14]

Being of lower socioeconomic status is often perceived as a barrier to having access to smart phones; however, Pew Research Center findings revealed that 65% of Blacks and 77% of Latinxs making less than $30,000 have either a smartphone or internet connection [6, 7]. According to the Pew Research Center, 72% of Blacks and 80% of Latinx have a smartphone or internet connection in the home [6, 7]. Additionally, greater than 70% of all Black and English-speaking Latinx with mobile phones use text messaging [13]. Black and Latinx are often more frequent users of mobile phones and report higher rates of text messaging than other racial/ethnic groups [13].

Chandler et al. (2019) examined the efficacy of a smartphone-enabled medication adherence program to improve medication adherence and blood pressure control [15]. The program titled, the Smartphone Medication Adherence Stops Hypertension (SMASH), enrolled 54 Latino adults in a 9-month randomized controlled trial [14]. Study participants were randomly assigned to the SMASH group (SMASH app, Bluetooth-enable blood pressure monitor, and electronic medication tray) or enhanced standard care group (SMS messages and short videos). The results indicated that the average systolic blood pressure was lower and medication adherence was higher in the SMASH group compared to the enhanced standard care group [14]. The SMASH app was an effective solution for promoting medication adherence among Latinx adults with uncontrolled hypertension [14]. The SMASH intervention provides important insights about the potential for using technology-based interventions among Latinx adults, but also highlights a need to explore the efficacy of other technology-based approaches for improving medication adherence in communities of color.

Lifestyle and behavioral interventions that incorporate the use of technology may be a promising and engaging approach for promoting hypertension self-efficacy and medication adherence among Blacks and Latinx. The primary objective of this mixed-methods study were to assess the feasibility of developing a lifestyle intervention that would assess hypertension management and the use of technology. The secondary objective was to identify barriers and facilitators of hypertension and explore preferences for future feasibility and lifestyle interventions among Blacks and Latinx living in a socioeconomically disadvantaged community.

## Methods

### Study Design

The study design was mixed-methods explanatory sequential. In a mixed-methods explanatory sequential design, the study begins with the collection of the quantitative data. Next, the quantitative data is analyzed, and the qualitative arm is designed informed by the results of the quantitative phase [16]. Data for the quantitative phase of the study were collected using a 43-item survey, followed by qualitative data collection of semi-structured individual interviews and a focus group. The quantitative phase of the study was conducted first and the protocol for the qualitative arm was refined based on the findings from the preliminary analysis of the quantitative data. The study was approved by the Institutional Review Board (IRB) at Penn State College of Medicine.

### Quantitative phase methods

#### Study recruitment

Study participants were recruited from a Federally Qualified Health Center (FQHC) located in Central, Pennsylvania, USA. The FQHC provides health care and resources to a racially and ethnically diverse community, as well as individuals with low socioeconomic status, uninsured, and vulnerable populations. Nurse educators and other health care providers discussed the study with their patients toward the end of their visit and referred interested patients to speak with the research staff on-site at the FQHC. The research staff reviewed the study details and the inclusion/exclusion criteria with the participants, after the materials were reviewed participants were verbally consented (*n*=30). Participants received a $20 gift card, as compensation for their time, they were not required to accept the gift card. Study recruitment and data collection was conducted from January to December 2017.

#### Inclusion and exclusion criteria

Eligible participants self-reported race/ethnicity as African American/Black (Hispanic/not Hispanic) or White (Hispanic), received primary care at the FQHC, were 21 years old or older, reported being diagnosed with high blood pressure, and were able to provide informed consent. Participants who spoke Spanish as their primary language were able to participate in the study assisted by a translator at the FQHC. Patients were excluded if they had cognitive or physical limitations that prevented them from providing consent or were not currently taking medications for hypertension.

#### Data collection

The 43-item survey included items assessing demographics, technology literacy, health literacy, and medication adherence. Data collection was conducted both in-person using REDCap or paper surveys, based upon the preferences of the participant. Data collected from the paper surveys were entered into REDCap by study staff and checked for accuracy by a data manager.

##### Technology literacy

Fourteen questions on the study survey were related to technology literacy. Questions were adapted from the 2012 Health Tracking Survey [17]. The survey consisted of questions including current cell phone or tablet usage and hypothetical questions regarding hypertension management through technology. These questions were assessed using the eHealth Literacy Scale (e-HEALS), which is an 8-item scale that measures knowledge, comfort, perceived skills, and accessing and applying electronic health information [17]. Sample questions from this section included: (a) Do you use the internet on a cell phone, tablet, or other mobile handheld devices, at least occasionally? (b) Do you ever look for health or medical information online? (c) Would you be willing to use your cellphone or tablet to take your blood pressure? and (d) Would you be willing to use your cellphone or tablet to take your blood pressure to remind you to take medication? All questions had two response options: no (0) or yes (1).

##### Medication adherence

Five survey items assessed medication adherence using questions from the 8-item Self Efficacy for Managing Medications and Treatments scale [18, 19]. The questions asked the patient to describe how confident they were in taking their medication. There were five response options ranging from “I am not confident at all” (0) to “I am very confident” (4).

### Statistical analysis

To answer the primary research questions, summary statistics, and frequency tables were examined. Data were analyzed using SAS Version 9.3.

### Qualitative phase methods

#### Study recruitment

Participants in the qualitative phase of the study were recruited from the FQHC, community health fairs, and during events taking place at a local Methodist church. The focus group lasted about an hour and the average time for the interviews was 10–15 min. Interviews were conducted over the phone or in-person depending upon the preference of the participant.

#### Data collection

The interview guide for the qualitative phase was refined based upon findings of the quantitative phase of the study. The questions used in the focus groups and interview questions were similar in scope and had minor adjustments to language to account for interviewing one person instead of a group interview. The interview guide included questions about hypertension, and managing antihypertensive medications, the use of technology to learn about medications and hypertension, national and community resources to facilitate hypertension management, and the design of future behavioral interventions to improve medication adherence and hypertension management. The focus group was led by the Principal Investigator (PI) and a Graduate Research Assistant. Interviews were conducted by the PI or by two doctoral level Graduate Research Assistants.

#### Data analysis

**Fig. 1:**
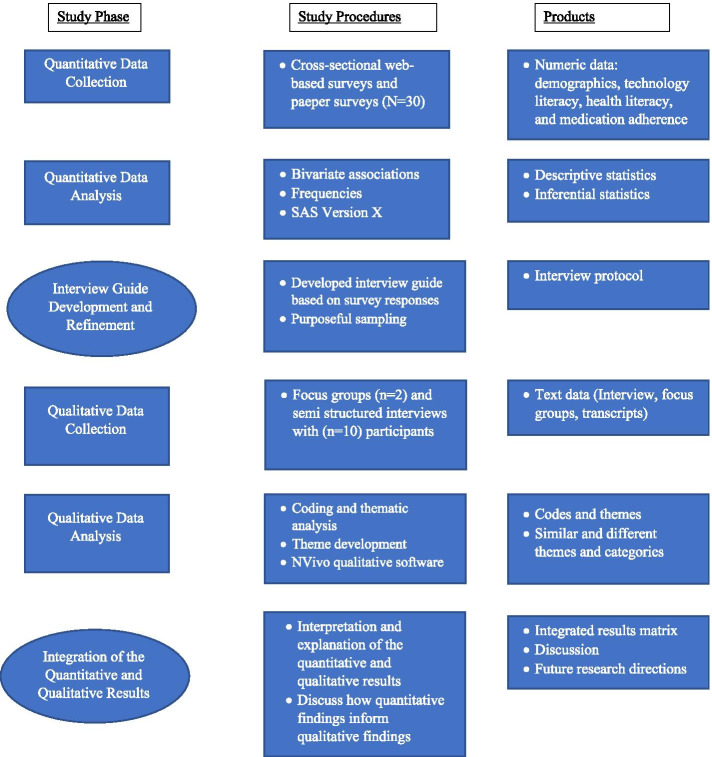
Explanatory sequential mixed-methods research design

The interviews and focus groups were audio recorded and transcribed by Rev.com. We conducted thematic analysis, by first reading two transcripts, making notes about themes and possible codes. The themes and codes were discussed as a group and edited as needed. We used inductive coding (see Fig. 1), and after refining the wording of codes and combining similar codes, we had nine codes and used the codes to identify key themes (Appendix). Transcripts were reviewed independently by two research team members, who created a codebook imported into NVivo 12. The study team members met to discuss disagreements and resolve questions, after resolving the disagreements, inter-rater reliability was moderate (*k* = 0.75).

## Results

### Quantitative phase

Table 1 presents the demographics of the 30 participants. Fifty-seven percent of the participants were female, and the mean age was 60.3 years. Fifty-nine percent identified as Black and 30% identified as Latinx. Forty percent never received a high school diploma, and most participants (76.7%) had a household income of less than $50,000. A little over half of the study participants (51.8%) were diagnosed between 6 and 15 years ago and 85.7% of participants visited their doctor “a few times per year” for hypertension.

**Table 1:** Demographics of 30 Black and Latinx participants

	*n*	%	Mean/range
**Gender**			
Male	13	43.3	
Female	27	56.7	
**Age, mean (years)**			60.3/(39–91)
**Race**			
American Indian or Alaska Native	1	3.5	
Black/African American	17	58.6	
White	4	13.8	
Prefer not to answer	7	24.1	
**Hispanic or Latinx**			
No	21	70.0	
Yes	9	30.0	
**Education level**			
Less than high school	12	40.0	
High school graduate	5	16.7	
Some college	4	13.3	
Trade/technical/vocational training	4	13.3	
College degree and above	5	16.7	
**Employment status**			
Employed	12	40.0	
Out of work	2	6.7	
Retired	10	33.3	
Unable to Work	6	20.0	
**Household income**			
Less than $50,000	23	76.7	
More than $50,000	3	10.0	
Unsure	4	13.3	
**Health status**			
Excellent	1	3.3	
Good	15	50.0	
Fair	9	30.0	
Poor	5	16.7	
**Years since hypertension diagnosis**			
Less than 1 tear	3	11.1	
1–5 years	6	22.2	
6–10 years	7	25.9	
11–15 years	7	25.9	
21 or more years	4	14.8	
**Doctor visits for hypertension**			
A few times per year	24	85.7	
Once per year	3	10.7	
Prefer not to answer	1	3.6	
**Number of medication taken for hypertension, mean**			2 (1–9)

#### Technology usage and literacy

Table 2 presents current technology usage among the participants. Out of 30 participants, 63.3% reported that they used the internet occasionally, 66.7% reported that they access the internet using a mobile handheld device, 73.3% had a cell phone, and 56.7% looked for health and medical information using a mobile handheld device.

**Table 2:** Technology usage and literacy among 30 Black and Latinx participants

	Yes, *n* (%)	No, *n* (%)
**Technology usage**		
1. Do you use the internet occasionally?	19 (63.3)	11 (36.7)
2. Do you access the internet using a cell phone, tablet, or other mobile handheld devices?	20 (66.7)	10 (33.3)
3. Do you have a cell phone?	22 (73.3)	8 (26.7)
4. Do you look for health or medical information online using a cell phone or tablet?	17 (56.7)	13 (43.3)
**Technology literacy**		
5. Would you take your blood pressure using a cellphone or tablet?	19 (63.3)	11 (36.7)
6. Would you use a cellphone or tablet to remind you to take medication?	17 (56.7)	13 (43.3)
7. Would you use a cellphone or tablet to record taking and forgetting medication?	15 (51.7)	14 (48.3)
8. Would you use a cellphone or tablet to learn about medication?	19 (63.3)	11 (36.7)
9. Would you use a cellphone or tablet to learn about hypertension?	16 (53.3)	14 (46.7)

Table 2 presents results from the technology literacy questions, which described helpful mobile device features when managing hypertension. Out of 30 participants, 63.3% reported they would take their blood pressure using a mobile handheld device, 56.7% would use a device to remind them to take their medication, 51.7% would use a device to record taking and forgetting medication, 63.3% reported that they would use a device to learn about medication, and 53.3% reported they would learn about hypertension using a handheld mobile device.

#### Medication adherence

Table 3 presents the findings related to medication adherence. Overall, participants reported being “very confident” about managing their medication. Of the 28 participants that answered the questions, 75% reported that they were very confident about managing their medication without help and very confident they would remember to take their medication as prescribed. Twenty-four participants responded to questions about using technology to manage medication, 54.2% were very confident that they could use technology to manage medication and treatment and 25% reported that they were not confident at all.

**Table 3:** Medication adherence among 30 Black and Latinx participants

	I am not confident at all, ***n*** (%)	I am a little confident, ***n*** (%)	I am somewhat confident, ***n*** (%)	I am quite confident, ***n*** (%)	I am very confident. ***n*** (%)
1. I can manage my medications without help, *n*=28	1 (3.6)		1 (3.6)	5 (17.9)	21 (75.0)
2. I can remember to take my medications as prescribed, *n*=28	1 (3.6)		1 (3.6)	5 (17.9)	21 (75.0)
3. I can use technology to help manage my medication and treatment, *n*=24	6 (25.0)	1 (4.2)	1 (4.2)	3 (12.5)	13 (54.2)

#### Qualitative phase

Figure 1 describes our process for using the quantitative data to inform the questions for the qualitative phase of the study. In our focus group, there were two participants and we conducted ten semi-structured interviews. The focus group was not well attended and after consultation with the health center medical director, we decided to shift to interviews. We had six men and six women participate in the qualitative phase of the study. We reviewed transcripts from the interviews and focus groups and used an iterative process of reviewing transcripts to modify and finalize our codes (Table 4). We identified four key themes: (1) importance of hypertension in the community, (2) barriers and facilitators of hypertension management, (3) self-efficacy in managing hypertension medication, and (4) future study design.

**Table 4:** Qualitative themes and codes

Themes	Codes
Importance of hypertension in the community	Community needs
Self-efficacy in managing hypertension	Health information seeking
	Knowledge about hypertension and medication adherence
	Use of technology for managing hypertension
Barriers and facilitators of hypertension management	Affording healthy foods
	Time
	Resources and support for managing hypertension
Future study design	Preferred setting and style
	Health topics of interest

##### Importance of hypertension in the community

Participants shared their perspectives on the importance of hypertension in the black community. Participants discussed topics ranging from the higher rates of high blood pressure in the Black community and they also highlighted the needs of Black people managing hypertension.

ID #: I3 Especially with blacks nowadays, a lot of us is dying from high blood pressure and having strokes..because people don’t know how serious the high pressure is.

ID#: I8 Especially in the black community, because those are the issues that needs to be addressed, you know, stress, high blood pressure. Number one is high blood pressure...It's more serious in our community.

##### Barriers and facilitators of hypertension management

Participants cited cost of healthy foods as “more expensive than regular food,” which was a key barrier to managing hypertension. Participants recognized that buying healthy foods is very important but expressed that people “have to buy what they can afford.” In addition to the cost of purchasing healthy foods, participants mentioned transportation challenges can make it difficult to access healthy foods, and the importance of having “something closer for folks that don’t have transportation.” Transportation challenges were not limited to just access but the ability to transport foods from the store to the home.

ID #: FG2 You got to remember; most people don't have money to buy that kind of food. And most of us buy like cold cuts and hamburgers, you know all that stuff shouldn't but now that we have the money, we can buy the fish and the turkey and all that good stuff.

ID#: FG1 But I think it'd be nice to have something closer for folks that don't have transportation because that's, that's also a challenge if you want to get down there. They have all the fruits and the vegetables and stuff, but you have to be able to get there and get home with that and not, you know, carry heavy bags with you.

Some participants expressed concerns about having a lack of resources in their community and mentioned they “don’t have a lot of resources.” Conversely, other participants felt that within their community there were resources that could be used to assist with hypertension management, but people may not be aware of those resources. Participants felt people “should take advantage of what their community provides.” They described local resources such as FQHCs, churches, and the Medical Outreach Program.

ID #: I8 A lot of people don’t have the exposure to being healthy because they don’t take the time out to go to a meeting. They’re not involved with the church … and they’re not in touch.

ID #: FG1We don’t have a lot of resources around our community…. That’s why we don’t want to help one another or it’s hard for you to go to somebody to ask certain things when you don’t have nothing.

##### Self-efficacy in managing hypertension medication

Participants were confident in their ability to manage antihypertensive medications, they reported taking their medications “first thing in the morning” and incorporated taking medications as a part of their daily “routine”. Participants shared their practices for remembering to take medication, such as “keeping it right in view” or taking the medications “after I eat my first meal.” Participants that expressed challenges remembering to take their medications cited forgetfulness or not wanting to take medications. Participants who struggled with forgetfulness expressed a need for tools that facilitate medication management.

ID #: I5 Oh yeah, absolutely. I keep it right in view. So, in the morning it’s right there on my chest (of drawers) in my bedroom, and I get up first thing in the morning, after I eat my first meal, I take my medication.

ID #: I8 I already know what needs to be done as far as taking my medication. I don’t forget, I'm not absentminded or anything of that nature. So, it’s like a routine, it’s regimental.

ID #: FG1 I’m not taking my medicine the way I should be. I just don’t do it sometimes.

##### Future study design from the perspective of participants

Participants described their preferences for future behavioral interventions for hypertension. While most of our study participants preferred in-person sessions, others reported that a mobile or technology intervention would more easily be integrated into their lives. They shared that an “app would probably be better” compared to in-person interventions. Others felt that an online format might work better and stated that “online you could just.. break it down in more detail” and it might help you “understand it a lot more.” Participants that did not use technology requested assistance and training to learn to use technology.

ID #: I3 I believe that online you could just... they break it down in more detail than you can in person. Because somebody can say something to you in person and you still didn’t really understand it and then you go online and then you read more about it and then online helps you understand it a lot more.

ID #: I4 So when you really talk to people face to face about it, and that might change some people minds instead of like going online. Like that’s might be one thing that you’ll talk about that you don’t want to read online. You could read it online, but until you meet that person that actually went through it, you won't believe it.

ID # I1They would have to show me how to use it because I don’t know how to use a computer.

Participants were also asked for the topics they would like to learn about and what would be helpful to incorporate into a behavioral intervention. Many participants described “exercise, dieting, and healthy foods” and stress as topics they would like to learn more about. The participants expressed a strong interest in having these topics included in the design of a future behavioral intervention.

ID # I9 I think it would be more fitness for me, yeah, my aspect would be more fitness a lot of it. Fitness and eating is probably my two biggest things.

ID # I7 Exercise, dieting, healthy foods. That’s a real big concern of mine right now, trying to come up with some different ways of trying to lose weight and exercise.

## Discussion

The objectives of this study were to assess the feasibility of developing a lifestyle intervention that would assess hypertension management and the use of technology. The secondary objective was to identify barriers and facilitators of hypertension and explore preferences for future feasibility and lifestyle interventions for Blacks and Latinx living in a socioeconomically disadvantaged community. In the quantitative phase of the study, participants reported high engagement with technology and were interested in using technology to manage hypertension. Over half of the participants self-reported high adherence to managing and remembering to take their medication as prescribed. The findings revealed participants were open to using technology to manage or learn more about hypertension. In the qualitative phase of the study, participants described their practices for managing hypertension and that it was often a routine practice for them, they also were split between preferring in-person vs. a technology-based intervention. Lastly, participants were interested in learning about health-related topics such as diet, exercise, and stress management.

The findings of our mixed-methods study were similar in scope to similar mHealth studies for chronic disease among minorities. Dick and colleagues (2011) conducted a pilot and feasibility mHealth study among 18 Black adults with diabetes [20]. Study participants engaged in an automated text messaging diabetes management program for 4 weeks. The authors found a decrease in missed medication doses and an increase in confidence in managing their condition after participating in the automated text-messaging program. A text messaging program might be an effective solution for promoting self-management of diabetes among Blacks [12]. Hacking et al. (2016) conducted a mixed-methods study to assess the impact of a mHealth intervention on knowledge, and health-related behaviors, and to understand participant experiences and reactions to the intervention [5]. Participants recruited from a hypertension clinic in Cape Town were randomly assigned to an intervention arm to receive short message services (SMS) or control arm (no SMS). The intervention did not significantly improve overall health knowledge, but the intervention arm reported higher knowledge scores on some questions about hypertension compared to the control arm. Participants preferred receiving health-related information on their phones rather than traditional health services or standard care of resources (e.g., pamphlet). Participants viewed the mHealth intervention (SMS) to motivate them to engage in healthy behaviors rather than a source for new information. Both studies highlight the potential impact of technology-based interventions for promoting healthy behaviors, and similar to our study provide insights to the preferences of the study participants.

Similar to other mixed-methods studies, our study had a small sample size, which limits the power and the ability to draw statistical inferences. Our study participants were recruited from Central Pennsylvania and may not be generalizable to other communities outside of Central Pennsylvania. There may have been issues of respondent bias, particularly respondents providing desirable answers to the interviewer. Medication adherence is typically over-reported on surveys, for the present study we were limited in using subjective measures. However, future studies may incorporate the use of subjective and objective measures. The qualitative study collected information that were self-reported by patients, and we only collected data at one time period; thus, we were unable to assess changes in medication adherence or use of technology. Additionally, the interviews and focus groups were conducted by the PI and three graduate research assistants, with varying levels of experience conducting qualitative research. The strengths of our study are that we captured the experiences and preferences for hypertension management and potential usefulness of a mHealth or eHealth intervention among Black and Latinx populations. Few studies have applied mixed-methods to explore both medication adherence and the use of technology for managing hypertension [21, 22]. Using a mixed-methods study design we elicited greater insights to medication adherence practices and interest in technology for a behavioral intervention, than using only quantitative methods. We also received useful feedback about local resources and gained knowledge of sources of information for members of the community.

The findings of this study contribute to the growing body of literature exploring the potential use of technology-based interventions among minorities and in a traditionally underserved community. The findings of this study could be used to guide the design of future interventions and programs tailored to meet the needs of Blacks and Latinx with hypertension. Specifically, interventions that apply novel approaches to sustain engagement in the management of hypertension over a lifetime not only for individuals reporting nonadherence but to provide continued support and engagement for individuals that typically report moderate to high adherence. Additionally, many of these interventions have not been available to minoritized communities and individuals living in socioeconomically disadvantaged communities. Thus, there are opportunities to adapt existing interventions and tailor them to the needs and preferences of the communities to make these same interventions available in communities that may carry a higher burden of chronic disease. Future studies led by this research team will explore opportunities for using technology to promote medication adherence, through reminders and health information about hypertension and hypertension medications, and to use technology to disseminate health messaging to provide study participants with connections to local and national resources to aid in the management of hypertension.

## Conclusion

This study supports the potential of developing a lifestyle and feasibility studies that uses technology as a tool for engaging racial/ethnic minorities and individuals living in socioeconomically disadvantaged communities in technology-based interventions, specifically for managing hypertension. Our study contributes to the literature by providing insights about the use of technology for managing hypertension among Blacks and Latinx. Study participants reported an interest in using technology for hypertension management and patient education. The findings will be used to inform the design of future lifestyle interventions by this research team; specifically, we will integrate technology-based reminders and the use of smartphones and tablets as a platform for delivering health information to individuals with hypertension. Lifestyle interventions that integrate technology to promote hypertension management might reduce multiple barriers to hypertension management and improve health outcomes.

## Data Availability

The datasets used and/or analyzed during the current study are available from the corresponding author on reasonable request.

## Appendix A

**Table 5:** A joint display highlighting quotes related to the medication adherence and technology the key themes of the surveys

**Medication adherence (MA) -** Medication adherence was influenced by side effects and beliefs about the effectiveness of the medication.	
Low MA scores	“At one point I wasn’t going to take the blood pressure medicine because of the side effects.”
High MA scores	“And then the medicine they gave me, actually works so I don’t be wanting to forget to take it.”
**Technological literacy (TL) -** Individuals expressed an interest in using technology to learn more about a hypertension management but training and guidance will be required for those that do not use technology regularly.	
Low TL scores	“They would have to show me how to use it because I don’t know how to use a computer.”
High TL scores	“I think an app would be great.”
	“I think the most, the most is the online thing. You find out more information and you just understand it better when you get it online.”
